# Molecular Phylogenetics of the genus *Hexachlamys (Myrtaceae)* using chloroplast and nuclear markers

**DOI:** 10.1186/1753-6561-5-S7-P6

**Published:** 2011-09-13

**Authors:** Fernanda Cruz, Andreia Turchetto-Zolet, Nicole Veto, Cláudio Mondin, Maurício Almerão, Rogério Margis

**Affiliations:** 1 Programa de Pós-Graduação em Genética e Biologia Molecular, Universidade Federal do Rio Grande do Sul (UFRGS), Brazil; 2 Centro de Biotecnologia UFRGS, Brazil; 3 Pontifícia Universidade Católica do Rio Grande do Sul, PUCRS, Brazil; 4 Centro de Biotecnologia UFRGS, Pesquisador CNPq, Brazil

## Background

Myrtaceae family includes more than 3,800 species of trees and shrubs, distributed mainly in tropical and subtropical regions of the world. Representatives of this family have great ecological significance for forest ecosystems, and are economically important species in the pharmaceutical, food, cosmetic and perfumery industry [1]. The genus *Hexachlamys* Berg. (Myrtaceae) has about 10 species distributed from the Southern to the Southeast of Brazil, and it also occurs in Paraguay, Argentina, Bolivia and Uruguay [2,3]. Since 1968 it has been considered an independent genus. It is distinguished morphologically from the genus *Eugenia* by pentamerous or hexamerous flowers and exserta radicle, and as many genera and species of the Myrtaceae family, it has a complex taxonomic classification [2,4,5]. The goal of this study was to conduct a molecular phylogenetic analysis among species of the genus *Hexachlamys* (Myrtaceae) using chloroplast (cpDNA) and nuclear (nrDNA) markers, and to verify its phylogenetic relationships with the genus *Eugenia* in order to contribute to the systematics and taxonomy of it.

## Methods

The samples were collected as leaf material(from herbaria species and samples collected in the field) of the genus *Hexachlamys* representing all described species, as indicated in herbariums and species of the *Eugenia* genus that occur in the Rio Grande do Sul state. Total genomic DNA was extracted using the CTAB method based on the protocol already described [6] and used for the PCR reactions. PCR products were sequenced on ABI PRISM 3100 sequencer (Applied Biosystem). Nucleotide sequences were aligned using CLUSTALW [7] implemented in MEGA5 (Molecular Evolutionary Genetics Analysis) version 5.0 [8], then checked visually and carefully improved manually before analysis. The phylogenetic analysis was reconstructed after nucleotide sequence alignments using three different approaches: the neighbor-joining (NJ), the Bayesian and the Maximum-Likelihood (ML) methods. The NJ, Bayesian and ML analysis were performed in MEGA 5.0, MrBayes [9] and PhyML [10], respectively.

## Results and conclusions

To date we obtained DNA sequences of 21 species of the genus *Eugenia* and seven species of the genus *Hexachlamys*. Universal primers that amplify chloroplast region (*trnL-trnF*, *trnL-intron*, *ycf5*, *accD*, *rbcLa*, *psbA-trnH*, *rpoB*, *rpoC1*, *ndhJ*, *matK* and *rps16*) and nuclear (*ITS*) were tested. The regions used for the phylogenetic analisys were chloroplast *accD*, *rpoB*, *rpoC1*genes and the nuclear *ITS*, since the other chloroplast regions were not possible to amplify and sequence in all sampled species. These cpDNA and nrDNA regions presented polymorphisms among species studied. Results from Bayesian, NJ and ML tree analysis produced similar topologies and revealed that *Hexachlamys* species did not form a monophyletic clade. The *Hexachlamys* species have grouped together *Eugenia* species with high bootstrap values, indicating that *Hexachlamys* can be a synonymous of genus *Eugenia.* These results corroborate with morphological data [11]. To confirm these results, other cpDNA and nrDNA markers will be tested and *Hexachlamys* species will be collected to substitute the species obtained in herbarium, since we found difficulties in the amplification of these species with all markers previously tested. This study can contribute to taxonomic classification of this group, as well as the field of conservation, since these species have an important economically and ecologically role.

Financial support: CNPq and FAPERGS
